# Exercise Improves Movement by Regulating the Plasticity of Cortical Function in Hemiparkinsonian Rats

**DOI:** 10.3389/fnagi.2021.695108

**Published:** 2021-06-14

**Authors:** Kaixuan Shi, Xiaoli Liu, Lijuan Hou, Decai Qiao, Yuan Peng

**Affiliations:** ^1^Department of Physical Education, China University of Geosciences, Beijing, China; ^2^College of Physical Education and Sports, Beijing Normal University, Beijing, China

**Keywords:** Parkinson's disease, exercise, motor cortex, local field potential, synchronization

## Abstract

Aberrant cortical spike-local field potential (LFP) coupling leads to abnormal basal ganglia activity, disruption of cortical function, and impaired movement in Parkinson's disease (PD). Here, the primary motor cortex mediated plasticity mechanism underlying behavioral improvement by exercise intervention was investigated. Exercise alleviates motor dysfunction and induces neuroplasticity in PD. In this study, Sprague-Dawley (SD) rats were injected with 6-hydroxydopamine (6-OHDA) to induce unilateral nigrostriatal dopamine depletion. Two weeks later, a 4-week exercise intervention was initiated in the PD + exercise (Ex) group. Multichannel recording technology recorded spikes and LFPs in rat motor cortices, and balanced ability tests evaluated behavioral performance. The balanced ability test showed that the total crossing time/front leg error/input latency time was significantly lower in PD + Ex rats than in PD rats (*P* < 0.05). Scalograms and LFP power spectra indicated increased beta-range LFP power in lesioned hemispheres, with exercise reducing LFP power spectral density. Spike-triggered LFP waveform averages showed strong phase-locking in PD motor cortex cells, and exercise reduced spike-LFP synchronization. Our results suggest that exercise can suppress overexcitability of LFPs and minimize spike-LFP synchronization in the motor cortex, leading to motor-improving effects in PD.

## Introduction

Parkinson's disease (PD) is a progressive neurodegenerative disease characterized by cell death of dopaminergic neurons in the basal ganglia (BG) (Kalia and Lang, 2015). Development of motor impairments, including bradykinesia, rest tremor, rigidity, and lack of coordination, have been associated with exaggerated synchronized oscillation of the beta band (13–35 Hz) in the corticostriatal circuit of patients with PD (Jankovic, 2008; Tinkhauser et al., 2018). These cortical beta oscillations, which may reflect active inhibition of movement and are related to maintenance of postural tone, are abnormally enhanced with dopamine (DA) depletion and coincide with the emergence of akinesia and bradykinesia (Pogosyan et al., 2009; Leventhal et al., 2012). Furthermore, after levodopa administration or deep brain stimulation (DBS), electrophysiological signal acquisition from the motor cortex and BG of PD patients shows reductions in this coherent beta frequency activity, with motor improvement (Kuhn et al., 2008; Babiloni et al., 2019; Wiest et al., 2020). Unfortunately, these pharmacological and surgical therapies often lead to side effects, including levodopa-induced dyskinesia (Huot et al., 2013; Antosik-Wojcinska et al., 2017).

Exercise can complement pharmacological therapies in these patients and has been used for rehabilitative management of PD since the 1990s (Duchesne et al., 2015; Pedersen and Saltin, 2015; Fox et al., 2018). While studies of exercise and physical therapy in PD have demonstrated clear improvements in motor performance, substantially less is known about the functional neuroplasticity resulting from long-term exercise interventions (Jakowec et al., 2016; Ferrazzoli et al., 2018). Thus, to explore the potential mechanisms underlying exercise-associated improvements in PD, cortical spikes and local field potentials (LFPs) were recorded simultaneously in control and lesioned rats (created by 6-hydroxydopamine [6-OHDA] injection into the medial forebrain bundle [MFB]), and spike-LFP relationships were investigated.

## Materials and Methods

### Animals and Experimental Procedures

Sprague-Dawley (SD) rats (male, 230–250 g, 8 weeks old, from Beijing Vital River Laboratory Animal Technological Company, Beijing, China) were housed in a controlled environment, with a 12/12-h light/dark cycle (lights on at 18:00) and *ad libitum* access to food and water. The Beijing Normal University Committee for Animal Care approved all experimental procedures, and this study complied with the guidelines set forth by the National Institutes of Health.

**Figure 1:**
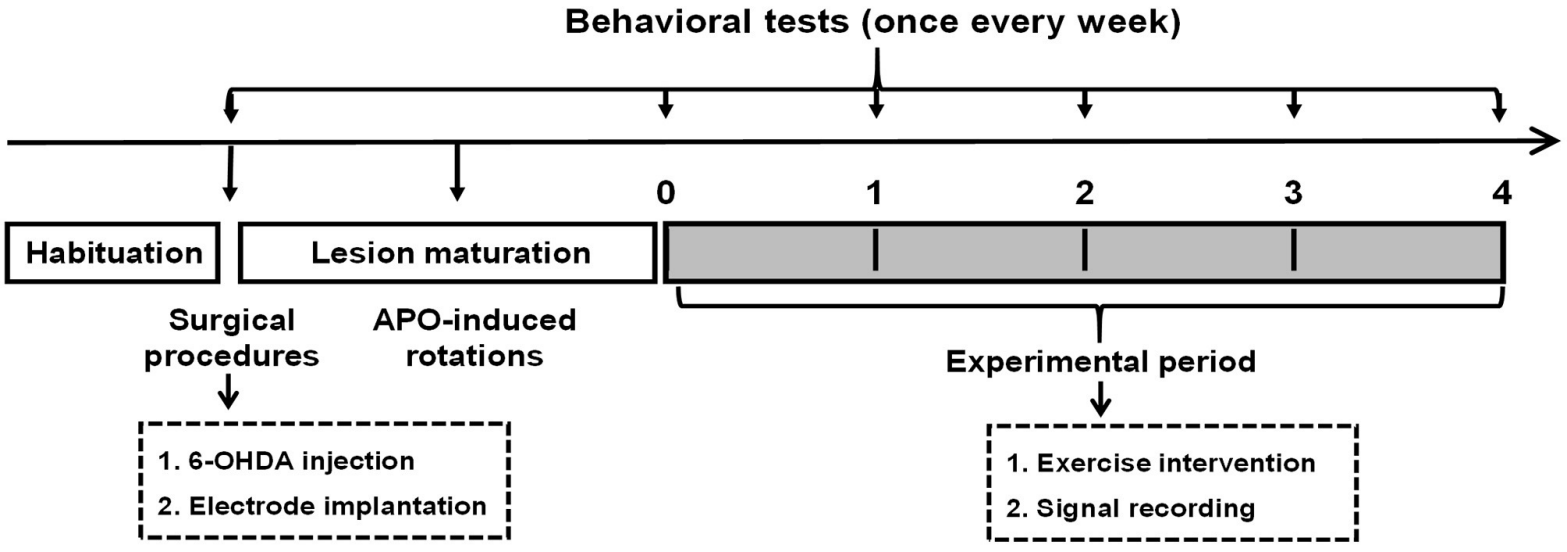
Experimental design. 6-OHDA, 6-hydroxydopamine.

All rats were initially randomly assigned to three groups: ➀ control group (*n* = 11), ➁ PD group (*n* = 14), and ➂ PD with exercise (PD + Ex) group (*n* = 14). As shown in Figure 1, rats in the PD + Ex group were placed on a treadmill, and all rats practiced using the automatic foot-fault equipment to familiarize them with the study procedures. The baseline behavioral level was evaluated after 1 week of habituation.

Then, animals in the control and PD groups (including the PD and PD + Ex groups) were administered vehicle or 6-OHDA, respectively. At 7 days after surgery, rats were tested for their rotational behavior using apomorphine (APO; 0.5 mg/kg, subcutaneous (s.c.); Sigma-Aldrich, St. Louis, MI, USA), with a total net number of rotations of ≥100 per 30 min indicating rats represented a model of Parkinson's disease, as described previously (Shi et al., 2017a). Subsequently, electrophysiological recording and automatic foot-fault testing were performed once per week. Starting during Week 1, rats in the PD + Ex group began a 4-week exercise intervention. At the end of Week 4, histological localization of electrodes and biochemical assays were performed.

### Nigrostriatal Lesions and Electrode Implantation

Each rat was anesthetized with chloral hydrate (350 mg/kg) and then placed on a stereotaxic frame (Stoelting, Chicago, IL, USA) fitted with atraumatic ear bars to level the skull in the dorsal-ventral plane (mouth bar was set at −3.3 mm). A small hole was made in the skull at the right MFB coordinate (Paxinos and Watson, 2007) (AP: −4.3 mm, ML: −1.5 mm, and DV: 7.6–7.8 mm), and an 8-μg solution of 6-OHDA (4 μL in total, per μL in 0.2% ascorbic acid and 0.9% saline, Sigma-Aldrich) was injected into this hole to lesion nigral dopaminergic cells, with an injection rate of 1 μL/min and slow syringe withdrawal (1 mm/min) over 5 min. Control animals received an equal volume of a solution of 0.2% ascorbic acid and 0.9% saline in the same manner.

Electrode implantation was conducted after 6-OHDA or vehicle injection. A chronic 16-channel microwire electrode array, which consisted of 4 × 4 rows of electrodes with an additional ground wire serving as a local reference (Stablohm 675, MicroProbes, USA; tip diameter = 35 μm, spacing = 300 μm), was implanted into the right primary motor cortex (M1) layers 5/6 (AP: 1.8~3.0 mm, ML: 2.3~3.3 mm, DV: 1.4–1.6 mm) (Paxinos and Watson, 2007). Finally, five stainless steel screws were anchored to the skull and wrapped with a reference wire. Dental cement (A-M Systems, WA, USA) was added to secure the skull screws.

### Exercise Protocol

Before undergoing surgery, all animals were adapted to the treadmill with a speed limit of 11 m/min. Two weeks after 6-OHDA injections, the sustained mandatory treadmill protocol (11 m/min, 30 min per day) was performed 5 days/week for 4 weeks (Tajiri et al., 2010; Chen et al., 2017) without electrical shock and at a 0° inclination. Meanwhile, rats in both the control and PD groups were placed on an unmoving treadmill for 30 min at a time.

### Behavioral Testing

To evaluate sensorimotor function, an automatic foot fault test was carried out before and after the 6-OHDA lesion was induced. One week before surgery, rats were placed at the departure box and trained to walk spontaneously across a horizontal scale with 77 bars to the arrival box. Rats underwent three training trials per day for 3 days, after which they could reliably walk from the departure box to the arrival box with fewer than five errors. Infrared beams and a sensor device (LocotronicH, Bioseb, Vitrolles, France; L × W × H: 128 cm × 28 cm × 20 cm) detected the movement of rats along the length of the ladder and were used to recognize and record the exact position and duration of errors in motor coordination (missteps or paw slips between any two rungs). The included software identified input latency times, front errors, and total crossing times. Each rat performed the test two times per day on Weeks 0, 1, 2, 3, and 4, with a rest of 5 min between two trials.

### Electrophysiological Data Acquisition and Analysis

The raw neural signals from each microwire of the electrode were amplified and recorded using the Cerebus^TM^ 128-Channel Data Acquisition System (Cyberkinetics Inc., Salt Lake City, UT, USA). Low-pass, filtered, LFP channels were amplified by 1000 ×, sampled at 1 kHz, and filtered under 250 Hz. Meanwhile, high-pass, filtered, waveform channels were amplified by 20,000 ×, digitized at 30 kHz, and bandpass-filtered between 250 and 5000 Hz. Spike detection and sorting were carried out automatically in real-time using the Centre software of the Cerebus^TM^ system. Simultaneously, the rat's automatic activities were tracked and recorded by the Neuromotive^TM^ system (Cyberkinetics Inc., Salt Lake City, UT, USA). Videotaped motor behavior was used to identify a sedentary behavioral state.

All recorded data were stored for additional offline analyses. Off-line Sorter software version 4.2.0 (Plexon, Dallas, TX, USA), Neuroexplorer software version 5 (Nex Technologies, Littleton, MA, USA), and custom-written MATLAB routines were used for these analyses.

#### Power Spectral Density Analysis of LFPs

Epochs of 300 LFP recordings (according to the rat's behavioral rest state) were captured, and the trapped filter was set to 50 Hz to remove power line interference and other high-frequency noises. Next, the spectrum value was normalized as the log of the raw power spectral density (PSD) from 0 to 80 Hz, which was constructed using a fast Fourier transform (FFT) with a based analysis and a 1-s moving window. The PSD and frequency blocks were set at 1,024 and 512 points at 0.2 Hz, respectively.

#### Spike Sorting and Cell-Type Classification

**Figure 2:**
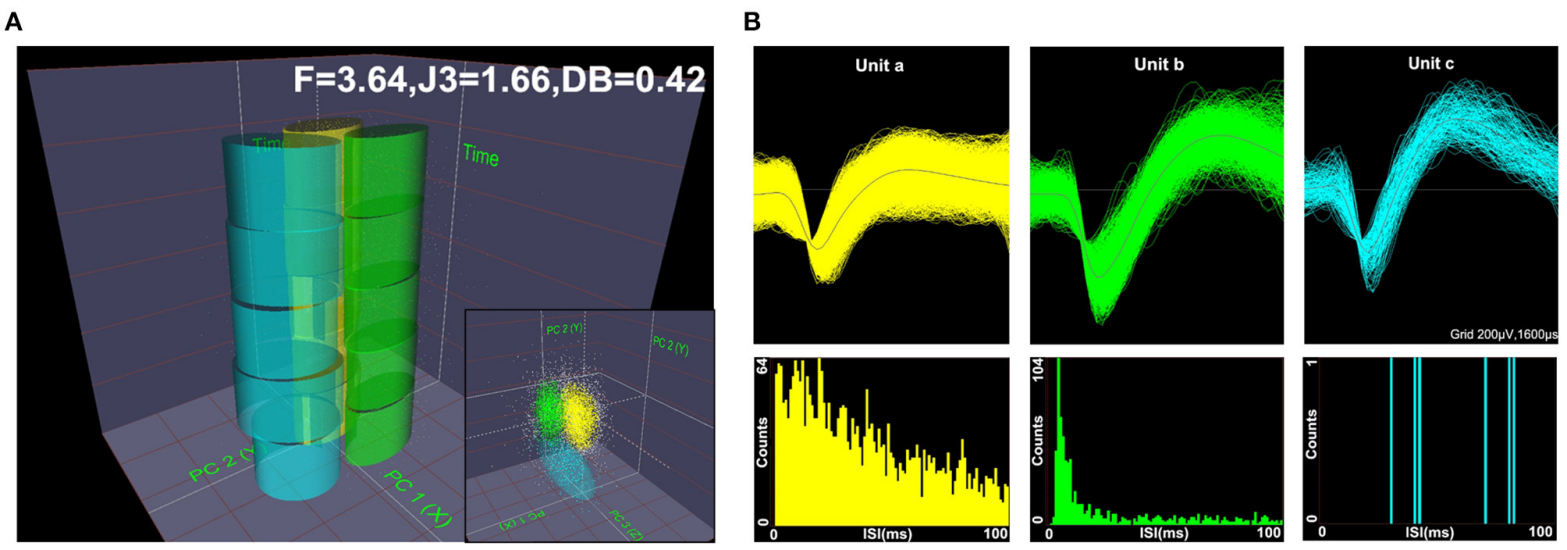
Diagram of offline unit sorting by principal component analysis (PCA) and the quality metrics of F, J3, and Davis-Bouldin (DB). Spikes were sorted using PCA in 2D and 3D PC spaces, **(A)** and the three units (a, b, c) were well-isolated based on their respective spike waveforms and interspike interval (ISI) histograms **(B)**.

Spike waveforms recorded from M1 were required to satisfy the following conditions: ➀ the threshold was three times the standard deviation; ➁ the signal-to-noise ratio was more than 2.5; and ➂ the interspike interval (ISI) histogram reflected spikes that did not occur within the assumed refractory period of 1.2 ms. Next, single-unit spikes were sorted using a principal component analysis (PCA) and a K-means cluster analysis to measure action potential widths and the frequency of spike trains. PC1 and PC2 (x and y axes) were plotted against time (z-axis) to isolate each unit in 2D and 3D PC space, as shown in Figure 2A. The F, J3, and Davis-Bouldin (DB) statistics were also calculated to determine whether the classification was qualified. Figure 2B shows the three identified units (a, b, and c) according to the spike waveform characteristics (top) and the ISI histograms (bottom).

Based on several studies comparing morphology with electrophysiological characteristics in M1, spikes were classified into two different neuronal subpopulations (Li et al., 2012; Lacey et al., 2014). One neuron type exhibited relatively long action potential widths (0.5–0.8 ms) and low discharge frequencies (<10 Hz) and were categorized as pyramidal neurons (PNs). Conversely, another neuron type had shorter action potential widths (0.2–0.5 ms) and higher spontaneous discharge frequencies (8–45 Hz) and was presumed to be interneurons (INs).

#### Spike-Triggered Waveform Analysis

**Figure 3:**
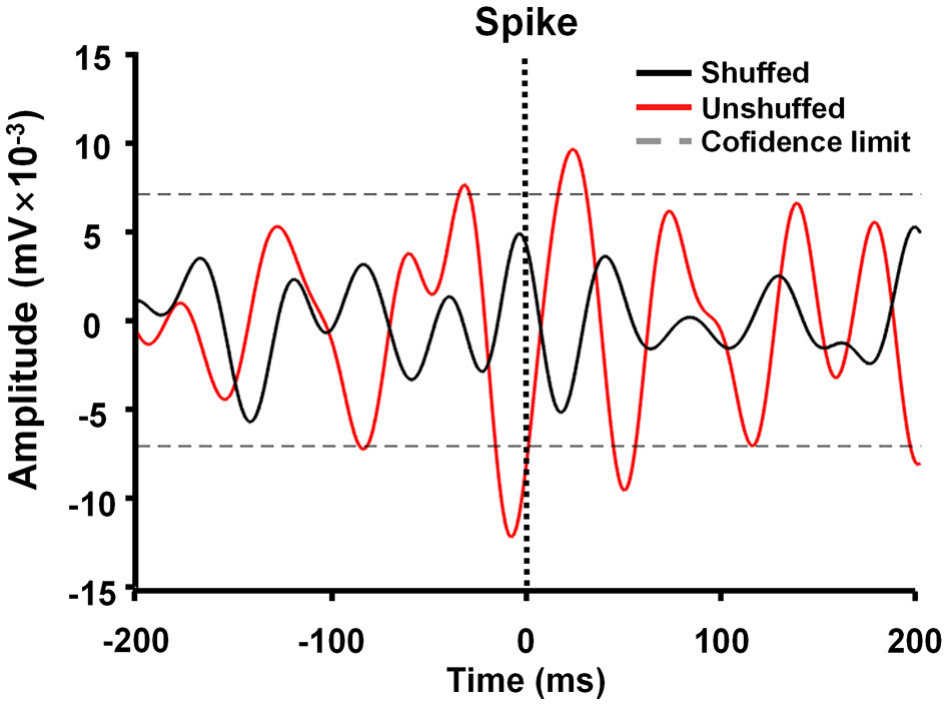
Example of M1 spike-triggered 10–30-Hz waveform averages. The unshuffled spike train (red) exceeded the 96% confidence interval of the shuffled spike train (black) and represented spike-local field potential (LFP) synchronization in the 10–30 Hz range.

Spike-triggered LFP waveform averages (STWA) were calculated to assess the degree of entrainment between spontaneous spike potentials of distinct neurons and LFP oscillations (Figure 3). LFPs were bandpass-filtered (beta band, 10–30 Hz) using the MATLAB signal-processing toolbox. STWAs were calculated as follows: 0 ms denoted the spike timestamp for a certain spike train, and filtered beta bands were averaged from two 200-ms epochs (unshuffled STWA). Subsequently, the original inter-spike intervals were randomly shuffled to produce different STWA. This process was repeated 1,000 times to provide a mean value for the beta band (shuffled STWA). Spikes were deemed to be related to beta oscillations when the unshuffled STWA exceeded the 96% confidence interval for the mean of the 1,000 shuffled spike trains at the timestamp (0 ms). Thus, the percentage of correlated neurons was calculated. The ratio of the mean unshuffled to the mean shuffled STWA was also calculated to estimate the proportion of synchronized neurons. When this ratio was close to 1, it indicated no phase locking between spikes and beta oscillations (Denker et al., 2011; Brazhnik et al., 2016; Wilson et al., 2018).

### Histology and Immunohistochemistry

After electrophysiological recordings were complete, the rats (control, *n* = 9; PD, *n* = 11; PD + Ex, *n* = 13) were deeply anesthetized by an intraperitoneal (i.p.) injection of chloral hydrate (450 mg/kg) and transcardially perfused with saline followed by 4% paraformaldehyde (PFA) in phosphate-buffered saline (0.1 M, pH 7.4). Brains were dissected and fixed in 4% PFA overnight at 4°C. Coronal sections were cut at M1 (50 μm), the substantia nigra pars compacta (SNc; 30 μm), and the striatum (30 μm) using a freezing microtome. The sections for recording electrode targets were mounted on glass slides and stained with cresyl violet. To assess dopaminergic denervation in PD models, sections from the SNc and striatum were analyzed by tyrosine hydroxylase (TH) immunohistochemistry, as described previously (Shi et al., 2019). The number of TH-immunoreactive neurons in the SNc and the optical density of TH-immunoreactive fibers in the striatum were evaluated. Results were normalized in each rat by values obtained on the right and left sides using the following formula: lesioned/unlesioned.

### Data Analysis

All data are presented as the mean ± standard error of the mean (SEM). Multiple comparisons were performed by one-way (before vs. after exercise intervention, i.e., “pre- vs. post” comparisons) or two-way (between two different groups) repeated-measures analysis of variance (ANOVA) followed by Student-Newman-Keuls *post-hoc* testing. All analyses were carried out using SPSS 21.0 (IBM Corp., Armonk, NY, USA) and Sigmaplot 13.0 (Systat Software, Inc, Richmond, CA, USA). A *P*-value of ≤ 0.05 was considered statistically significant.

## Results

### Behavioral Results

**Figure 4:**
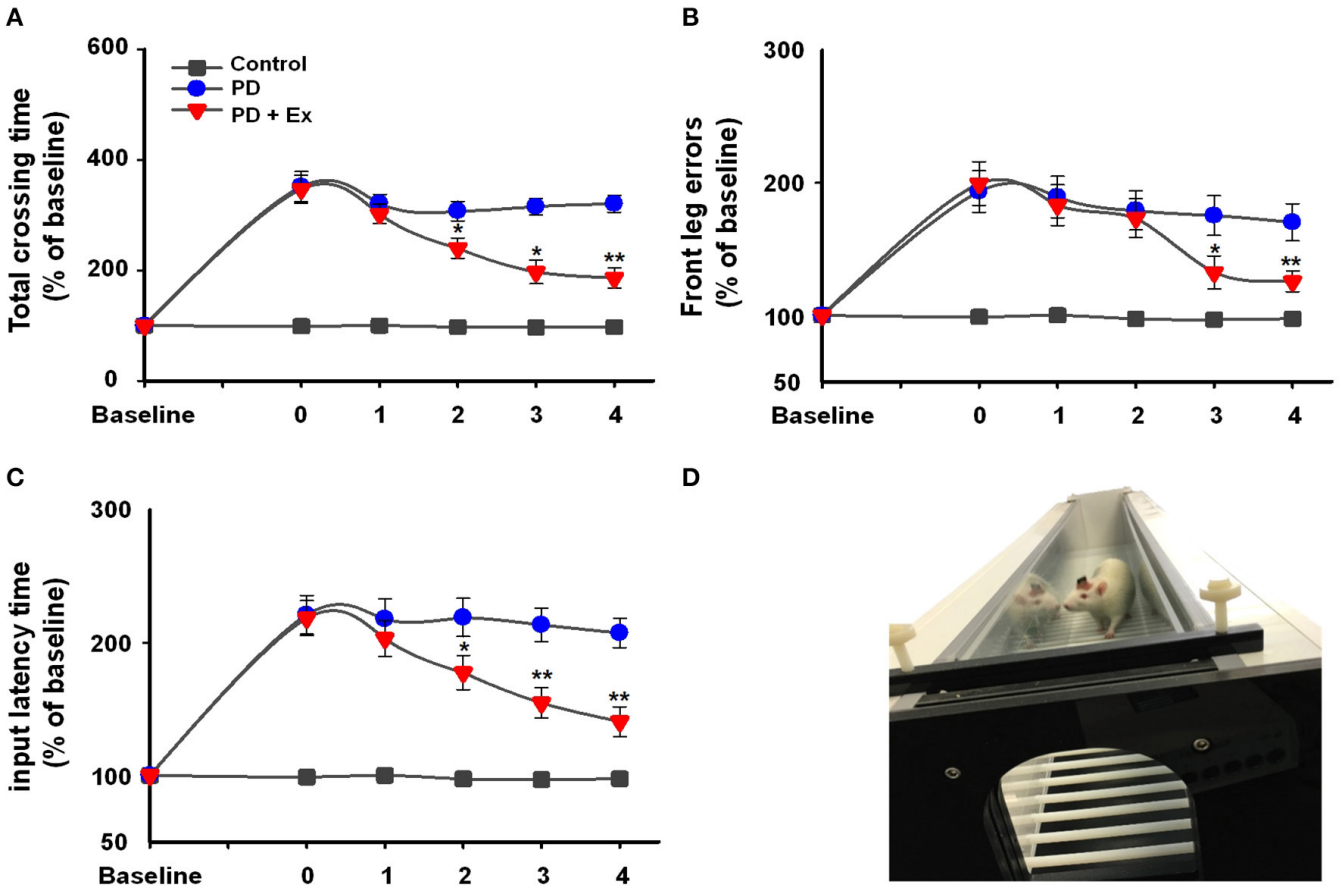
Automatic foot fault test **(D)** results in the control, Parkinson's disease (PD), and PD + exercise (Ex) groups. Time course of the automatic foot test and the percentages of baseline for total crossing time **(A)**, front leg error **(B)**, and input latency time **(C)**. Compared with the Week 0 standard *: *P* < 0.05, **: *P* < 0.01.

Rats were placed on the automatic foot fault device (Figure 4D), and 6-ODHA-lesioned rats were shown to have deficits in the adjusting step test. In the control and PD groups, the three test indicators showed no significant changes compared to Week 0 (*P* > 0.05, Figures 4A–C). For the total crossing time (% baseline), exercise improved the performance of PD rats by enhancing vigor and reducing the duration of time required to cross the beam at Weeks 2, 3, and 4 (at Weeks 2 and 3, *P* < 0.05; at Week 4, *P* < 0.01, Figure 4A) compared to Week 0. Input latency times showed the same trend (at Week 2, *P* < 0.05; at Weeks 3 and 4, *P* < 0.01, Figure 4C), whereas deficits in front leg errors were reduced at Week 3 (*P* < 0.05) and remained relatively stable at Week 4 (*P* < 0.01).

### Histological and TH-Immunoreactivity Results

APO-induced rotation showed that 24 rats were successfully modeled in the PD and PD + Ex groups, with net rotations consistent with the criterion for the PD model (165.43 ± 17.67 r/30 min > 100 r/30 min). Failed modeling occurred in four rats that had net rotations <60 r/30 min.

**Figure 5:**
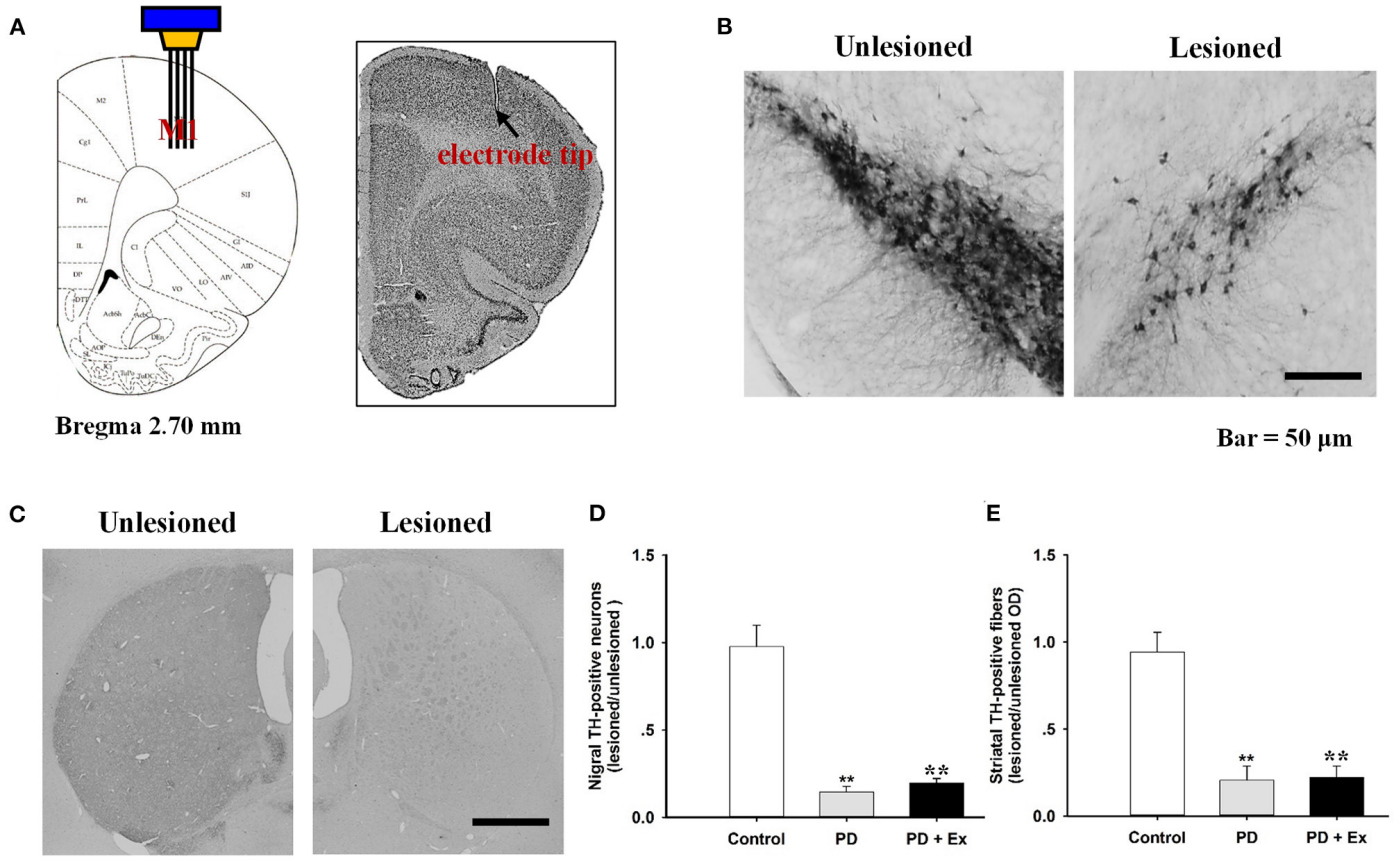
Histological results in the M1 and tyrosine hydroxylase (TH)-immunostaining results in the substantia nigra pars compacta (SNc). The recording electrode site at M1 was confirmed on the coronal section and adapted from Paxinos and Watson (2007) **(A)**. TH-immunostaining results in the SNc **(B)** and striatum **(C)** from both unlesioned and lesioned sides. There were significantly lower numbers of TH-positive neurons in the Parkinson's disease (PD) and PD + exercise (Ex) groups than in the Control group (**: *P* < 0.01); however, there was no significant difference between the PD and PD + Ex groups (*P* > 0.01) **(D,E)**.

Histological staining with cresyl violet confirmed correct placement of the recording electrode array in M1 (layer 5) on coronal sections (Figure 5A). Figures 5B,C shows immunostaining for TH in rat SNc and striatum, with obviously lower numbers of TH-immunoreactive cells and fibers on the lesioned side relative to the unlesioned side. Compared with the control group, 6-OHDA-lesioned rats had significantly reduced nigrostriatal projections in both the PD and PD + Ex groups (*P* < 0.01). The exercise intervention was unable to significantly reverse the nigrostriatal depletion in these groups (*P* > 0.05, Figures 5D,E).

### Time-Dependent Changes in Spontaneous Firing Rates in M1 Correlates With the Efficacy of Exercise

**Figure 6:**
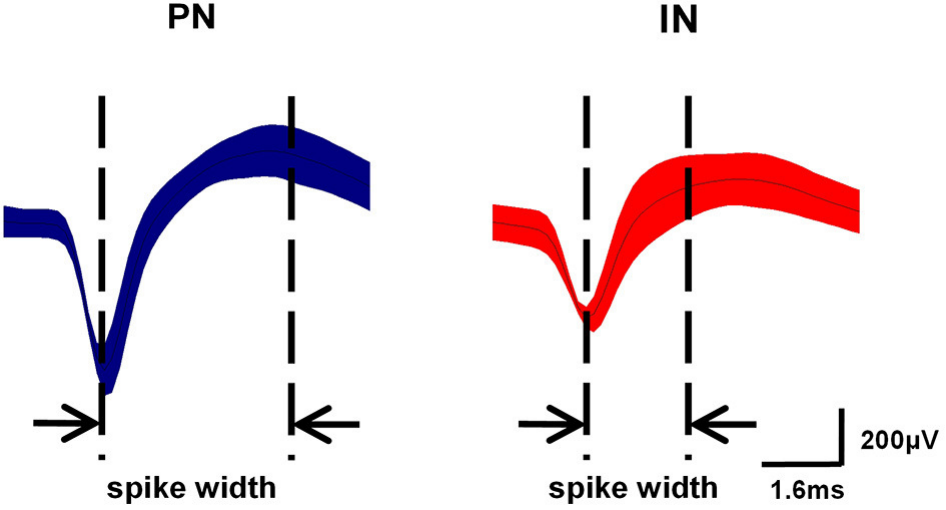
Classification of neurons in M1. Waveform examples of pyramidal neuron (PN) (blue) and interneuron (IN) (red) subtypes in M1. PNs exhibited relatively long action potential widths (0.5–0.8 ms) and low spontaneous firing rates (<10 Hz), while INs had shorter action potential widths (0.2–0.5 ms) and higher spontaneous firing rates (8–45 Hz).

As described in section Spike Sorting and Cell-Type Classification, PNs and INs have distinct electrophysiological characteristics. Based on the electrophysiological properties of spontaneous firing rate and spike width (Figure 6), PNs and INs can be distinguished explicitly. The proportions of these two types of neurons in the study groups are summarized in Table 1.

**Table 1:** Proportions of pyramidal neurons (PN) and interneurons (IN) in the motor cortices of rats in the control, Parkinson's disease (PD), and Parkinson's disease + exercise (PD + Ex) groups.

**Group**	**Total neurons**	**PN (%)**	**IN (%)**
Control (*n* = 9)	113	86 (76.1%)	27 (23.9%)
PD (*n* = 11)	147	104 (70.7%)	43 (29.3%)
PD + Ex (*n* = 13)	156	123 (78.8%)	33 (21.2%)

**Figure 7:**
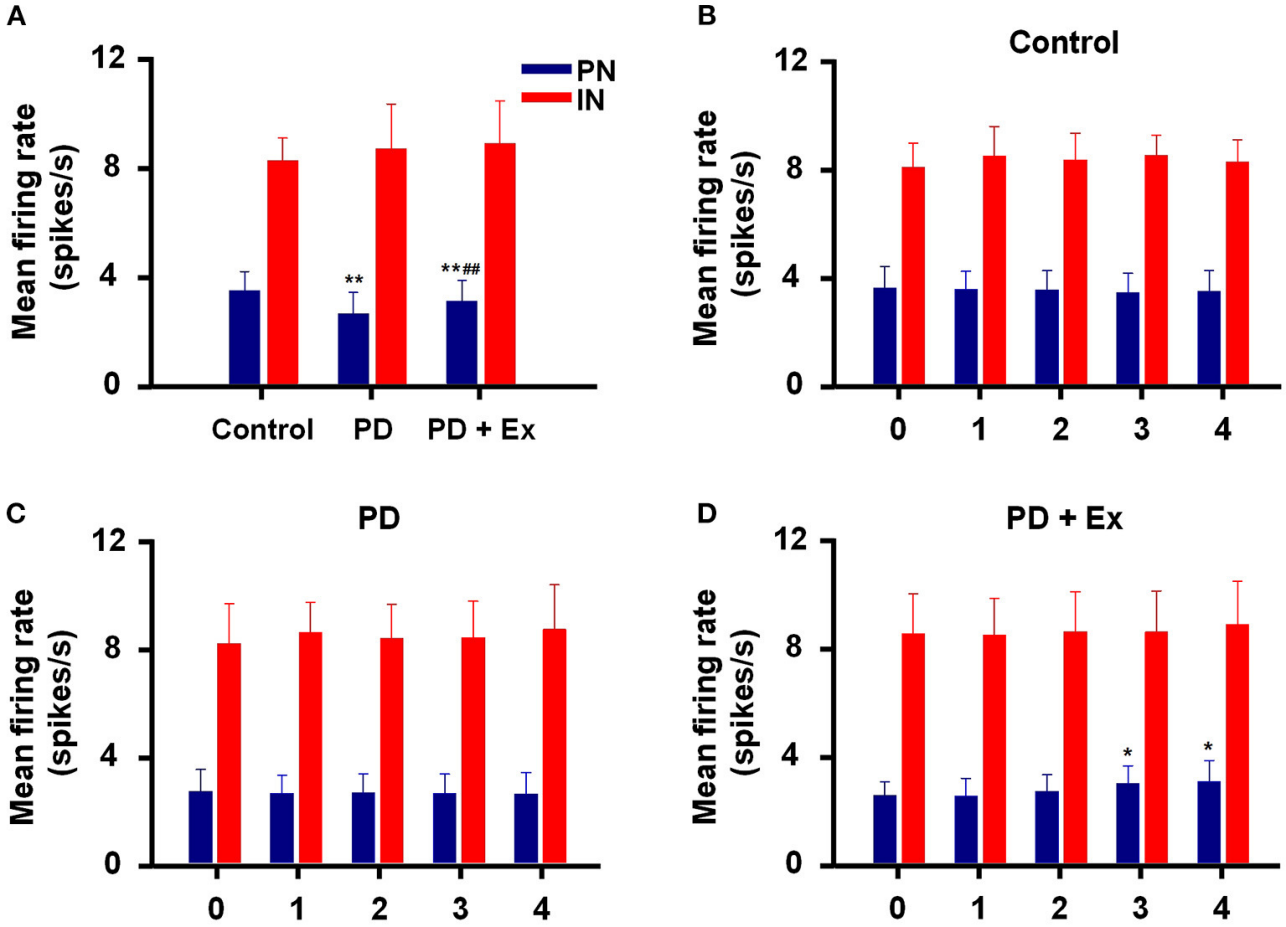
Inter- and intragroup comparisons of the mean firing rates of pyramidal neurons (PNs) and interneurons (INs) in M1. The intergroup analysis of PNs (blue) and INs (red) at Week 4 is shown in **(A)**. The mean firing rates of PNs and INs varied with time in the Control **(B)**, Parkinson's disease (PD) **(C)**, and PD + exercise (Ex) **(D)** groups. Compared with the Control group or Week 0 data, *: *P* < 0.05, **: *P* < 0.01; and compared with the PD group, #: *P* < 0.05, ##: *P* < 0.01.

An intergroup analysis of the spontaneous firing activity of PNs and INs at Week 4 indicated that the mean firing rate was different after induction of 6-ODHA lesions. As shown in Figure 7A, the mean firing rate of PNs was significantly lower in the PD group than in the control group (3.427 ± 0.703 vs. 2.569 ± 0.79 Hz, *P* < 0.01). Compared with the PD group, the mean frequency of PN firing in the PD + Ex group was obviously increased (3.033 ± 0.77, *P* < 0.01) but was still significantly lower than in the Control group (*P* < 0.01). The average frequency of firing for INs, however, did not significantly differ between the three groups (*P* > 0.05).

Intragroup comparisons of mean firing rates showed that these two types of neurons did not have significant alterations at Weeks 0, 1, 2, 3, or 4, either in the control group or PD group (*P* > 0.05, Figures 7B,C). For the PD + Ex group, however, the mean firing rate of PNs was significantly increased from Week 3 to 4 compared with Week 0 (*P* < 0.05), though there were no changes in INs during exercise training (*P* > 0.05; Figure 7D).

### Exercise Attenuates Cortical Beta Oscillatory LFP Power in 6-ODHA-Lesioned Rats

**Figure 8:**
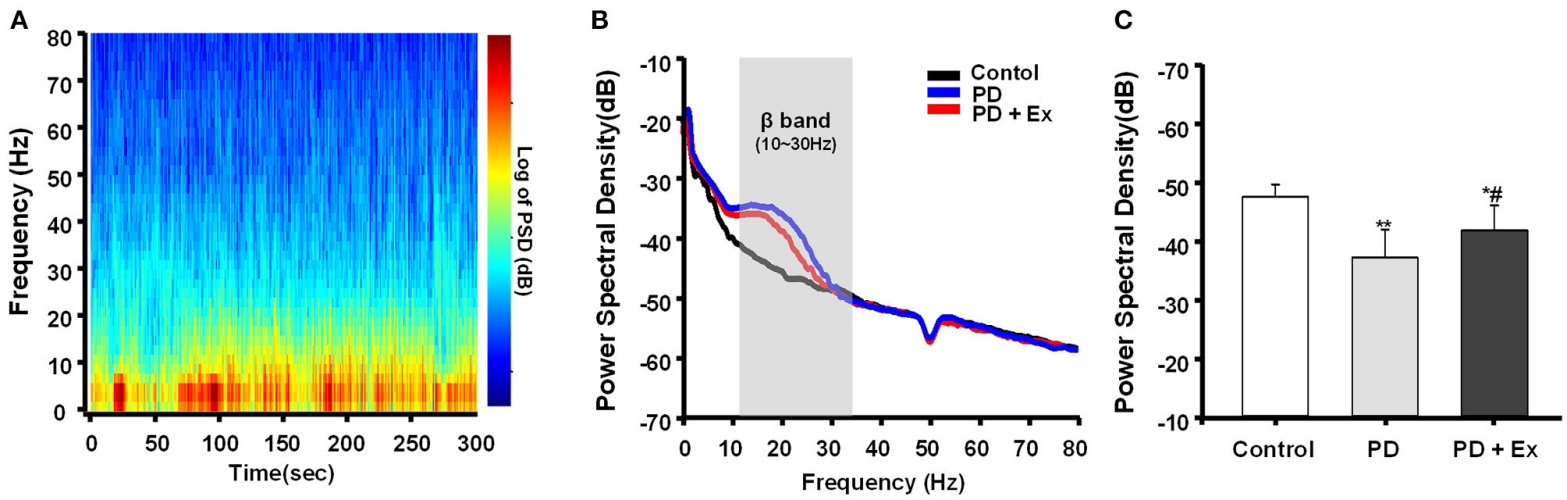
Power spectral density (PSD) of β local field potentials (LFPs) recorded in M1. A representative fast Fourier transform (FFT)-based spectrogram depicts the time-frequency spectral power of motor cortex LFPs during 5-min epochs of rest **(A)**. Linear graphs show averaged LFP power (0–80 Hz) spectra for the Control, Parkinson's disease (PD), and PD + exercise (Ex) groups **(B)**, with the gray area showing that the beta band in the 10–30 Hz range of LFP power was significantly increased in 6-hydroxydopamine (6-OHDA)-lesioned rats. The average PSDs of the three groups are shown in **(C)**. The PSD of the beta band was increased in the PD group compared to the Control group. For the PD + Ex group, the PSD of the beta band was decreased compared to the PD group but was still higher than in the Control group. Compared with the Control group, *: *P* < 0.05, **: *P* < 0.01; and compared with the PD group, #: *P* < 0.05.

In this study, hemiparkinsonian rats (PD and PD + Ex groups) exhibited higher cortical LFP power in the 10–30 Hz range than control rats in the resting state (*P* < 0.01 and *P* < 0.05, respectively, Figures 8A,B). The PSDs of beta LFPs were significantly higher in the PD + Ex group than in the PD group (*P* < 0.05, Figure 8C), and this change was time-dependent, which has been confirmed in our previous study (Shi et al., 2017b).

### Exercise Disrupts the Pathological Synchrony Between M1 Neurons and Beta Oscillations

**Figure 9:**
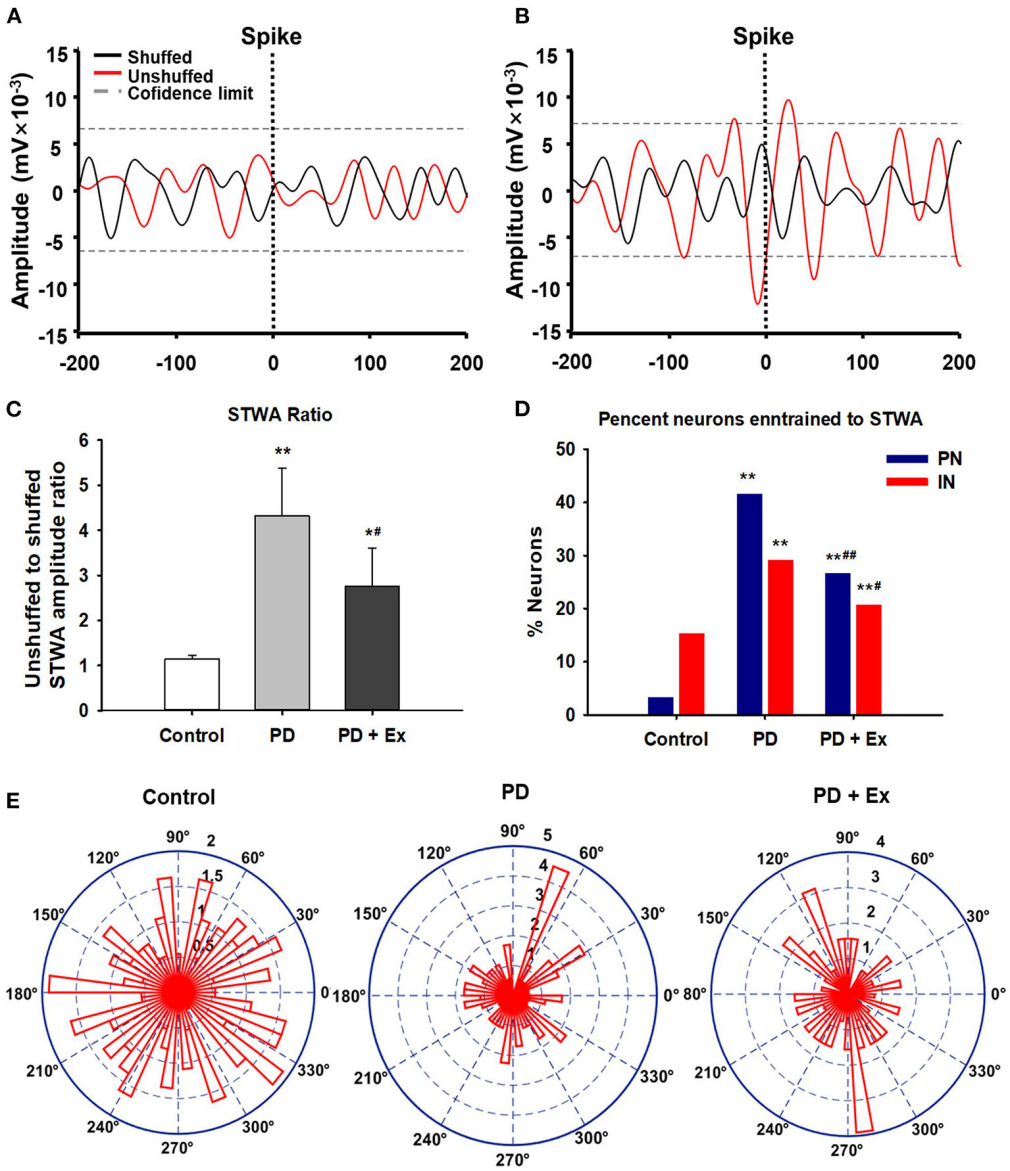
Phase locking of cortical spiking with 10–30 Hz local field potentials (LFPs) in the three groups. Unsynchronized spike-triggered LFP waveform averages (STWA) **(A)**. Example STWA for a typical cortical spike train paired with an LFP in the 10–30 Hz range **(B)**. The ratio of STWA-based unshuffled-to-shuffled spike trains indicating cortical spike-LFP synchronization **(C)**. Bar graphs show the proportions of pyramidal neurons (PNs) and interneurons (INs) significantly synchronized with 10–30 Hz LFP oscillations **(D)**. To assess STWA, mean phase angles between spikes and cortical LFP oscillations were significantly clustered around different phases of cortical 10–30 Hz LFP oscillations, as the polar plots show in **(E)**. Compared with the Control group, *: *P* < 0.05, **: *P* < 0.01; and compared with the PD group, #: *P* < 0.05; ##: *P* < 0.01.

The spike–LFP relationship was examined in M1 to determine whether changes in spike timing correlated with the increases in LFP beta-range (10–30 Hz) activity observed in 6-OHDA-lesioned rats. As described in section Data Analysis, increases in the LFP power in the 10–30-Hz range in the lesioned hemisphere have been associated with an increased synchronization of spikes and LFP oscillations in the same frequency range. Figure 9A shows STWA without obvious phase locking, and Figure 9B depicts typical STWA synchronization. The mean ratios of unshuffled-to-shuffled STWA peak-to-trough amplitudes (STWA ratios) of cortical neurons in the 10–30-Hz band were compared between the three groups. Figure 9C shows that the STWA ratio was higher in PD models than in control rats (compared to the control group, PD: *P* < 0.01, PD + Ex: *P* < 0.05). Following the exercise intervention, phase-locking of spikes with beta-band oscillations was reduced in the PD + Ex group compared to the PD group (*P* < 0.05). In the control group, PNs and INs did not show notable synchronization of spiking with the beta band. The percentage of neurons (both PNs and INs) entrained to STWA was significantly increased in PD model rats compared to control rats (compared to the control group, percent PNs and INs in PD: *P* < 0.01, percent PNs and INs in PD + Ex: *P* < 0.01). After the exercise intervention, the percentage of these neurons was smaller than in the PD group (percent PNs: *P* < 0.01; percent INs: *P* < 0.05; Figure 9D). The polar plots in Figure 9E show the distribution of STWA phases from spike trains in the M1 with cortical LFP 10–30-Hz oscillations. The phase angles between spikes and beta oscillations appeared random in control rats; however, the STWA of spike trains in PD rats showed strong phase-locking at about 60°. The STWA of cortical neurons in the PD + Ex group seemed to be relatively scattered but still presented with obvious phase-locking consistent with the STWA ratio results.

## Discussion

Previous studies have demonstrated that exercise training can modulate activity in the cortical motor areas of PD patients, as well as in animal models of parkinsonism (Wang et al., 2015; Alberts et al., 2016; Ginanneschi et al., 2021). In this study, multichannel electrode arrays were implanted into the M1, and spike trains of neurons and LFPs were recorded and analyzed in hemiparkinsonian rats during a therapeutically effective exercise intervention. This approach allowed us to address several key questions related to the role of functional changes in M1 on PD, providing insights into the mechanism of exercise-induced neuroplasticity.

This study identified increases in spiking discharges and beta-band oscillations, which was consistent with the results of previous studies conducted in humans and PD animal models, including rodents and non-human primates (Hammond et al., 2007; Zhuang et al., 2018; Holt et al., 2019). Abnormal spike and beta LFP activity at various levels of the cortical-basal ganglia-thalamocortical circuit have been observed in PD individuals (patients and animal models), though the origin of these changes remains unknown (Goldberg et al., 2004; Dejean et al., 2008; Stein and Bar-Gad, 2013). It is generally hypothesized that pathological changes in the motor cortex may contribute to the motor dysfunction observed in parkinsonian conditions. Li et al. (2012) have confirmed that exaggerated spike trains and beta oscillations can deteriorate motor control and lead to the development of akinesia and bradykinesia. Furthermore, several studies focusing on the mechanisms of subthalamic nuclei DBS or L-3, 4-dihydroxyphenylalanine (L-DOPA) treatment have shown that L-DOPA administration reduces the cortical power in the beta range and that STN-DBS induces reductions in the synchronization of oscillations in the cortex-basal ganglia circuit.

Although M1 is a crucial basal ganglia output area that transforms neurophysiological signals into motion instructions, there have only been a few previous studies related to exercise-induced plasticity of cortical neural activity (e.g., electrophysiological evaluations) at the cellular level in PD patients (Bonavita, 2020; Feng et al., 2020). Wang et al. (2013) analyzed regional cerebral blood flow-related tissue radioactivity in three-dimensionally reconstructed brains by statistical parametric mapping to explore the neural substrates underlying exercise-based neurorehabilitation. The results of that study highlighted exercise-dependent functional reorganization both in the motor (M1) and limbic circuits (dorsal striatum). In this study, we also found direct evidence of neural plasticity in M1 cells in PD rats following exercise induction.

Our results confirmed exaggerated beta-range LFP activity in cortical cells of 6-ODHA-lesioned rats, which was effectively eliminated by exercise. Exercise was also able to increase the mean firing rate of PNs; however, the mean firing rates of INs and all cells were not significantly changed. A few factors may have contributed to this result. First, firing rates of PNs and INs vary in different animal models and with different signal acquisition methods (Degenetais et al., 2002; Xu and Baker, 2018). The majority of previous studies have identified a reduced firing rate in PNs without obvious changes in INs, which may reflect a compensatory change after DA depletion (Pasquereau and Turner, 2011; Shepherd, 2013). Another possibility is that exercise-induced improvements in motor function might modulate the degree of coupling between spikes and LFP oscillations. Multiple studies have shown that spike trains tend to be phase-locked to overactive beta bands in various nuclei and motor cortices (Brazhnik et al., 2012, 2014). In this study, we used multichannel recording technology to simultaneously record spikes and LFPs, which allowed us to analyze the level of synchrony between them. The results showed that the STWA ratio and the percentage of neurons were increased in 6-OHDA-lesioned rats, contributing to movement disorders. Exercise likely induces a critical reduction in the synchronization of spike and beta-range oscillations in M1.

Exercise modalities such as treadmill training, Tai Chi, tango dancing, boxing, and forced cycling incorporating both motor skill learning and aerobic training; they may work synergistically to facilitate neuroplasticity and functional restoration to drive motor and cognitive behavioral improvement in PD. Treadmill can be easily adjusted for exercise elements (include speed and gradient) and its training in subjects with PD has been proved in multiple studies to promote behavioral performances (Da Silva et al., 2018; Dauwan et al., 2021). Forced treadmill and wheel running approaches used to drive movement improvement in patients with PD and finding in animal studies that support the potential mechanism (Ferrazzoli et al., 2018). It should be noted that the dopaminergic depletion of the nigrostriatal system was unable to be reversed by the exercise intervention, as has previously been reported (Sconce et al., 2015; Hood et al., 2016; Shi et al., 2019). This finding might be attributed to the fact that the exercise intervention was started 14 days after the 6-OHDA injection, at which point DA depletion may have been completed. Moreover, the behavioral improvements appeared after 2 or 3 weeks exercise intervention. This time-dependence on training might due to the PD rats being in the advanced stage, which affects the cognitive elements and needs more repetitively practice to restoration. Despite the 6-OHDA-induced injury severity, other factors also influence exercise-mediated neuroprotection, including the exercise initiated time, the exercise intervention administrated pre or at mild stage of PD may facilitate neuroprotection or an alternative process for behavioral recovery involve neuroplasticity (Jakowec et al., 2016; Ferrazzoli et al., 2018).

Indeed, numerous studies have shown that chronic exercise cannot reverse dopaminergic cell death and that improvements in motor performance are related to other compensatory mechanisms (O'Dell et al., 2007; Churchill et al., 2017; Hou et al., 2017). Moreover, studies with PD animal models have shown that exercise decreases excessive striatal glutamate content, restores glutamatergic receptors, and inhibits glutamate-mediated excitotoxicity, thereby contributing to a functional reorganization of the corticostriatal pathway (Van Leeuwen et al., 2010; Toy et al., 2014). Thus, it is likely that the efficacy of the exercise intervention was dependent on plastic changes in the glutamate-mediated corticostriatal pathway, which affected the activity of cortical neurons and led to changes in motor symptoms.

In conclusion, this study provides evidence that a treadmill exercise administration can effectively alleviate motor deficits in PD rats. This exercise-induced behavioral improvement involved the disruption of abnormal neural activity in the M1 region of PD rats. The synchronization of spike firing and beta oscillations in this model may hold the key to this process, and the functional consequences of this synchrony on exercise-induced neuroplasticity in PD rats need to be verified.

## Data Availability Statement

The raw data supporting the conclusions of this article will be made available by the authors, without undue reservation.

## Ethics Statement

The animal study was reviewed and approved by Beijing Normal University Committee for Animal Care.

## Author Contributions

XL, KS, and DQ: conceived and designed the experiments. KS and LH: performed the experiments. KS, LH, and YP: analyzed the data and wrote the paper. All authors contributed to the article and approved the submitted version.

## Conflict of Interest

The authors declare that the research was conducted in the absence of any commercial or financial relationships that could be construed as a potential conflict of interest.
